# Dental disorders are associated with minimal synovial changes in the temporomandibular joint in horses

**DOI:** 10.3389/fvets.2026.1810777

**Published:** 2026-06-30

**Authors:** Joao Ricardo Kunz, Rubens Peres Mendes, Milena Carol Sbrussi Granella, Adson Costa, Rodrigo Romero Corrêa, Anderson Fernando de Souza, Joandes Henrique Fonteque, Silvio Kau-Strebinger

**Affiliations:** 1 Departamento de Medicina Veterinária, Centro de Ciências Agroveterinárias, Universidade do Estado de Santa Catarina (Udesc), Lages, Brazil; 2 Departamento de Cirurgia, Faculdade de Medicina Veterinária e Zootecnia, Universidade de São Paulo, São Paulo, Brazil; 3 Unit of Morphology, Department of Biomedical Sciences and Pathobiology, University of Veterinary Medicine Vienna, Vienna, Austria

**Keywords:** arthrocentesis, arthropathy, cytology, equine dentistry, odontoplasty

## Abstract

**Introduction:**

Dental disorders have been proposed as factors contributing to temporomandibular joint (TMJ) discomfort in horses, but objective evidence linking these conditions to synovial fluid alterations remains limited. This study evaluated whether the severity of dental abnormalities influences the physicochemical and cytological characteristics of TMJ synovial fluid and whether occlusal adjustment induces measurable synovial changes over time.

**Methods:**

Thirty-three horses were assigned to mild, moderate, or severe dental abnormality groups. Synovial fluid was collected bilaterally from the discotemporal joint compartment before and 30, 60, and 90 days after dental treatment. Physicochemical and cytological variables were compared among severity groups and sampling times.

**Results:**

Synovial fluid characteristics remained stable across most variables, groups, and time points. A clearer synovial fluid appearance was observed in the contralateral TMJ of horses with severe dental abnormalities at 60 and 90 days, whereas an increased synovial fluid volume was detected in the ipsilateral joint of horses with moderate dental abnormalities at 90 days. No other variables differed significantly (*p* > 0.05).

**Discussion and conclusions:**

Dental disorders and their correction produced limited measurable effects on synovial fluid composition in the equine TMJ, indicating substantial biochemical stability. These findings suggest that, despite the proposed association between dental abnormalities and TMJ dysfunction, synovial fluid physicochemical and cytological characteristics remain largely unaffected by dental abnormality severity and occlusal adjustment.

## 1 Introduction

The Temporomandibular Joint (TMJ) is the primary joint responsible for mastication in horses and plays an essential role in the biomechanics of prehension and grinding of feed (1). It is a diarthrodial, condylar joint composed of two connected cavities (discomandibular and discotemporal compartments) separated by a biconcave fibrocartilaginous disc and filled with synovial fluid (2, 3).

Although TMJ-related disorders are considered relatively uncommon, they are likely underdiagnosed because their clinical signs often overlap with those of dental disease (4). Reported clinical manifestations include dysmastication, localized swelling, pain in palpation and manipulation, behavioral changes, bony deformities, progressive weight loss, and dental malocclusions (4). Despite these signs, TMJ abnormalities are a less frequent cause of poor performance (5). The variability in clinical presentation and the absence of standardized diagnostic pathways further complicate recognition of TMJ disorders (6). Diagnostic investigation is challenging and relies on history, clinical examination, and ancillary tests, particularly imaging combined with synovial fluid analysis (7, 8).

Synovial fluid is a plasma dialysate responsible for joint lubrication and metabolic cellular support. By altering mandibular biomechanics, tooth loading patterns, and TMJ dynamics (9), dental malocclusions may disrupt cellular TMJ homeostasis. Such disturbances can result in synovial changes ranging from viscosity alterations to modifications in inflammatory mediators (10). Synovial fluid evaluation is a clinically valuable tool to characterize intra-articular processes, differentiate between septic and non-infectious arthritis, and monitor therapeutic responses (11). Understanding how dental disorders interact with TMJ synovial characteristics is therefore relevant for clinicians aiming to interpret joint health, treatment outcomes, and functional recovery more accurately.

The aim of this study was to evaluate how different severities of dental abnormalities influence the physicochemical and cytological characteristics of the TMJ synovial fluid in horses, and to assess the effects of occlusal adjustment over time. We hypothesized that: (i) greater severity of dental abnormalities would be associated with more pronounced alterations in synovial fluid; (ii) occlusal adjustment would lead to progressive normalization of synovial parameters and; (iii) changes would be more evident in the TMJ ipsilateral to the most affected dental arcade.

## 2 Methods

Thirty-three client-owned horses (15 mares and 18 geldings), with a mean age of 13.3 ± 7.7 years and no history of previous dental treatment, were included in the study. All animals first underwent a clinical examination (12), followed by sedation with detomidine hydrochloride (0.02 mg/kg, IV). After sedation, each horse was subjected to bilateral arthrocentesis of the discotemporal compartment of the TMJ, followed by a complete oral cavity examination. The discotemporal compartment was selected for synovial fluid sampling due to its larger size and the higher likelihood of successful fluid collection, allowing a more reliable and consistent arthrocentesis procedure.

**Figure 1:**
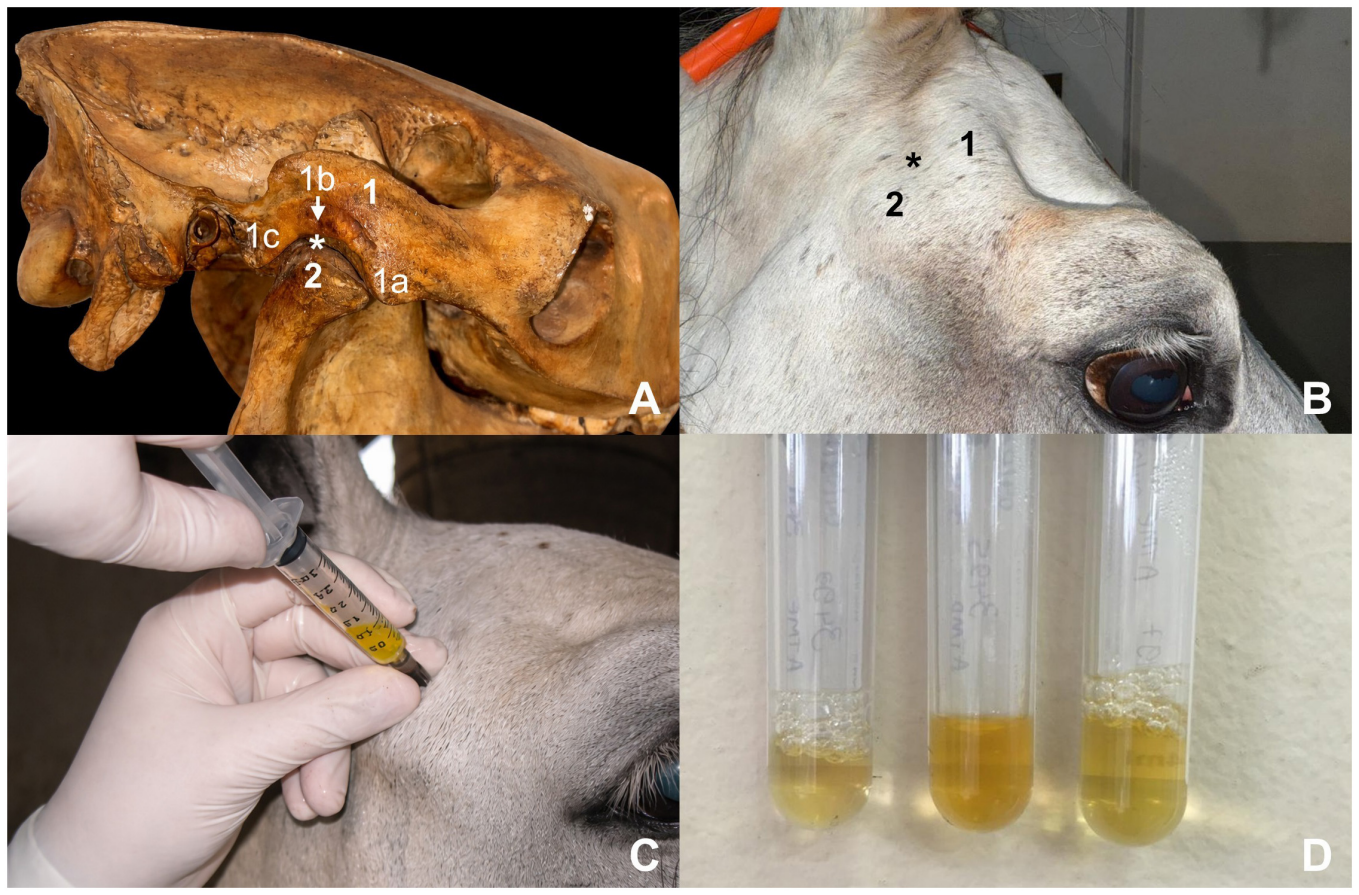
Anatomical landmarks for arthrocentesis of the temporomandibular joint to obtain synovial fluid from the discotemporal compartment. Landmarks are illustrated in a skeletal specimen **(A)** and in a live horse **(B, C)**. Synovial fluid collected in tubes containing 10% EDTA for physicochemical and cytological analysis **(D)**. 1: temporal bone (1a, rostral articular tubercle; 1b, rostral mandibular fossa; 1c, caudal retroarticular process); 2: mandibular condyle. The asterisk indicates the needle insertion site.

The TMJ region was aseptically prepared (13). Arthrocentesis was performed following the technique described by Rosenstein et al. (14). The mandibular condyle was palpated midway between the lateral canthus of the eye and the base of the ear, and the zygomatic arch was identified dorsally. The puncture site was selected 0.5–1 cm ventral to an imaginary line joining these landmarks, at the point of least resistance to palpation (Figure 1A). A 30 × 0.7 mm needle attached to a 5-mL syringe was inserted in a rostroventral direction and advanced 1.5–3 cm. Synovial fluid was collected from both TMJs of each horse and immediately transferred to tubes containing 10% EDTA anticoagulant (Figure 1B).

Samples were assessed for appearance, volume, color, viscosity, total protein, pH, glucose concentration, and cellularity (15). Synovial fluid volume was determined as the maximum amount recovered during arthrocentesis. Viscosity was assessed at the time of sample collection, as previously described by Steel (16). The pH, glucose concentration, and occult blood in synovial fluid were measured using Combur-Test reagent strips (Roche Diagnostics, Basel, Switzerland). The physicochemical variables were coded to: appearance: clear = 1; slightly turbid = 2; moderately turbid = 3; intensely turbid = 4. Occult blood: negative = 0; trace = 1; + = 2; ++ = 3; +++ = 4.

Total protein concentration and density were measured using a handheld refractometer (ATTAGO Co), and erythrocyte and nucleated cell counts were performed using a hemocytometry method. Cytology was obtained from sediment after centrifugation (3500 rpm for 3 min), and differential cell counts were performed on rapid panoptic stain slides (8).

Following synovial fluid collection, a complete dental examination was performed (1). Horses were classified into mild, moderate, or severe dental abnormality groups according to the criteria of Salem et al. (17) and Dixon et al. (18): *Mild*: sharp enamel points without mucosal ulceration, non-pulpal fractures, and diastemata without gingival inflammation. *Moderate*: sharp enamel points or focal malocclusions (hooks, ramps, or steps < 10 mm) causing mucosal lesions; diastemata with grade 1 periodontal disease (0/4); diagonal bites with 2–3° inclination; grade 1 infundibular caries (0/4). *Severe*: dental fractures with pulp exposure; diagonal bites >4°; malocclusions >10 mm with mucosal ulceration; diastemata with periodontal disease ≥ grade 2 (0/4); grade ≥2 infundibular caries (0/4). Dental evaluations and synovial fluid sampling were repeated at 30, 60, and 90 days following occlusal adjustment. All oral examinations and classification of dental abnormalities were performed by one author (JRK) with more than 10 years of experience in equine dentistry.

For analytical purposes, the ipsilateral TMJ was defined as the joint corresponding to the side with the most pronounced dental abnormalities, while the contralateral joint referred to the opposite side. In cases of bilateral or generalized dental changes, classification was based on the side presenting the most severe alterations.

Data distribution was assessed using the Shapiro-Wilk test. Parametric variables (pH, glucose concentration, total protein concentration, and total nucleated cell count) were analyzed using repeated-measures ANOVA (RM-ANOVA) followed by Tukey's *post hoc* test. Non-parametric variables (appearance) were analyzed using the Friedman test with Dunn's *post hoc* test and Bonferroni-adjusted *p*-values. The analyses were performed using the Sigmaplot program (version 12.5) and differences were considered statistically significant when probability values were less than 5% (*p* < 0.05).

## 3 Results

### 3.1 Sampling

A total of 33 horses were included in the study, comprising 11 in the mild group, 12 in the moderate group, and 10 in the severe group. The number of successful arthrocentesis procedures varied across groups and time points. Considering bilateral sampling, in the mild group (*n* = 22 joints), successful synovial fluid collection was achieved in 17 joints at baseline, 18 at 30 days, 13 at 60 days, and 19 at 90 days. In the moderate group (*n* = 24 joints), successful collections were obtained in 24 joints at baseline and 30 days, 21 at 60 days, and 15 at 90 days. In the severe group (*n* = 20 joints), successful sampling was achieved in 18 joints at baseline, 20 at 30 days, and in 10 and 14 joints at 60 and 90 days, respectively.

### 3.2 Physical properties

Changes in synovial fluid volume were observed in the ipsilateral TMJ to the dental abnormalities. Horses in the moderate dental abnormality group showed an increase in synovial fluid volume at 60 and 90 days compared with baseline (*p* < 0.05; Table 1). However, mean volume values in the mild and severe groups also varied over time, although these differences did not reach statistical significance (*p* > 0.05), and considerable dispersion among individual animals was observed.

**Table 1:** Mean values and standard deviations (x̄ ± SD) of synovial fluid variables from the temporomandibular joint ipsilateral to the dental abnormalities in 33 horses before and after dental treatment, divided into mild (*n* = 11), moderate (mod, *n* = 12), and severe (sev, *n* = 10) groups.

Variables	Group	Time points			
		Baseline	30 days	60 days	90 days
Volume (mL)^*^	Mild	1.07 ± 0.68	1.26 ± 0.70	1.18 ± 0.63	0.97 ± 0.53
	Mod	0.90 ± 0.34b	1.06 ± 0.59ab	1.08 ± 0.68a	1.46 ± 0.49a
	Sev	1.04 ± 0.48	1.03 ± 0.25	1.02 ± 0.32	1.38 ± 0.74
Appearance	mild	3.00 ± 1.41	1.55 ± 0.82	1.89 ± 1.17	2.50 ± 1.35
	Mod	1.58 ± 1.08	1.83 ± 1.19	1.67 ± 1.07	1.22 ± 0.44
	Sev	2.44 ± 0.73	2.10 ± 1.45	2.00 ± 1.22	2.67 ± 1.03
Occult blood	Mild	3.40 ± 0.84	2.30 ± 1.77	2.78 ± 1.64	3.40 ± 1.07
	Mod	2.42 ± 1.56	2.09 ± 1.51	2.45 ± 1.63	1.67 ± 1.80
	Sev	1.78 ± 1.72	2.80 ± 1.32	2.80 ± 1.64	2.83 ± 1.47
Density	Mild	1.024 ± 0.005	1.025 ± 0.004	1.027 ± 0.007	1.022 ± 0.002
	Mod	1.026 ± 0.005	1.028 ± 0.007	1.026 ± 0.006	1.025 ± 0.002
	Sev	1.026 ± 0.005	1.026 ± 0.003	1.029 ± 0.007	1.026 ± 0.004
pH	Mild	7.45 ± 0.76	7.64 ± 0.64	7.69 ± 0.65	7.70 ± 1.21
	Mod	7.96 ± 0.75	7.71 ± 0.54	7.85 ± 0.75	7.61 ± 0.49
	Sev	7.94 ± 0.92	7.75 ± 0.59	7.50 ± 0.50	7.93 ± 0.53
Glucose (mg/dL)	Mild	1.67 ± 0.87	1.36 ± 0.50	1.43 ± 0.53	1.40 ± 0.52
	Mod	1.67 ± 0.71	1.42 ± 0.51	1.60 ± 0.52	1.22 ± 0.44
	Sev	1.43 ± 0.79	1.43 ± 0.79	1.20 ± 0.84	1.50 ± 0.55
Total protein (g/dL)	Mild	3.37 ± 1.16	3.11 ± 1.04	3.80 ± 1.22	3.12 ± 1.46
	Mod	3.23 ± 0.67	3.58 ± 0.58	3.34 ± 0.99	2.88 ± 0.46
	Sev	3.20 ± 0.96	3.49 ± 0.68	4.10 ± 1.25	3.08 ± 0.86

Asterisk (^*^) indicates a variable with statistical differences, where the mean value followed by different lowercase letters indicates *p* < 0.05 among time points (rows). No statistically significant differences were detected among groups (columns) (*p* > 0.05).

In the contralateral joint, a significant improvement in synovial fluid appearance was identified in horses with severe dental abnormalities, characterized by reduced turbidity at 60 and 90 days relative to baseline (*p* < 0.05; Table 2). No significant changes in appearance were detected in the mild or moderate groups (*p* > 0.05). Synovial fluid color and viscosity did not differ between groups or across time points in either joint (*p* > 0.05); however, both variables showed considerable dispersion among individual animals.

**Table 2:** Mean values and standard deviations (x̄ ± SD) of synovial fluid variables from the temporomandibular joint contralateral to the dental abnormalities in 33 horses before and after dental treatment, divided into mild (*n* = 11), moderate (mod, *n* = 12), and severe (sev, *n* = 10) groups.

Variables	Group	Time points			
		Baseline	30 days	60 days	90 days
Volume (mL)	Mild	0.94 ± 0.79	0.98 ± 0.78	0.74 ± 0.71	0.73 ± 0.45
	Mod	0.92 ± 0.62	0.98 ± 0.48	0.88 ± 0.57	0.85 ± 0.75
	Sev	0.93 ± 0.58	0.92 ± 0.28	0.39 ± 0.46	0.63 ± 0.73
Appearance^*^	Mild	2.18 ± 1.66	2.00 ± 1.67	1.00 ± 1.00	1.73 ± 1.19
	Mod	2.45 ± 1.50	2.09 ± 1.04	1.36 ± 1.03	1.27 ± 1.01
	Sev	2.80 ± 0.92a	2.20 ± 1.23ab	1.30 ± 1.57b	1.10 ± 1.37b
Occult blood	Mild	2.45 ± 1.97	3.09 ± 1.578	1.55 ± 1.81	1.73 ± 1.85
	Mod	2.92 ± 1.44	1.83 ± 1.70	1.75 ± 1.55	1.75 ± 1.60
	Sev	3.20 ± 1.40	2.70 ± 1.49	1.70 ± 2.00	1.60 ± 1.90
Density^*^	Mild	0.74 ± 0.48	0.56 ± 0.54B	0.65 ± 0.52	0.75 ± 0.48
	Mod	0.85 ± 0.39	0.94 ± 0.30A	0.86 ± 0.40	0.68 ± 0.50
	Sev	0.82 ± 0.43	0.82 ± 0.43AB	0.52 ± 0.54	0.62 ± 0.53
pH	Mild	5.45 ± 3.51B	6.20 ± 3.30	5.09 ± 4.06	5.54 ± 3.62
	Mod	8.20 ± 0.94Aa	6.83 ± 2.21a	6.83 ± 3.23a	4.96 ± 3.69b
	Sev	7.90 ± 0.61AB	7.50 ± 0.41	3.95 ± 4.21	4.05 ± 4.30
Glucose (mg/dL)	Mild	1.09 ± 0.94	1.18 ± 0.75	0.91 ± 0.83	1.00 ± 0.78
	Mod	1.18 ± 0.87	1.55 ± 0.69	1.36 ± 0.67	1.09 ± 0.94
	Sev	1.00 ± 0.94	0.80 ± 0.79	0.70 ± 0.95	0.40 ± 0.52
Total protein (g/dL)^*^	Mild	2.27 ± 1.55	1.59 ± 1.54B	2.21 ± 2.02	2.15 ± 1.78
	Mod	2.79 ± 1.40	3.33 ± 1.18A	2.86 ± 1.53	2.00 ± 1.51
	Sev	2.51 ± 1.53	2.66 ± 1.64AB	1.97 ± 2.14	2.41 ± 2.33

Asterisk (^*^) indicates a variable with statistical differences, where the mean value followed by different lowercase letters indicates *p* < 0.05 among time points (rows) and different uppercase letters indicates *p* < 0.05 among groups (columns).

### 3.3 Chemical characteristics

In the contralateral TMJ, horses in the moderate group exhibited higher synovial fluid density and total protein concentration at 30 days compared with the mild group (*p* < 0.05; Table 2). At baseline, synovial fluid pH was also higher in the moderate group compared with the mild group. Within this group, pH values at 90 days were significantly lower than at the other time points (*p* < 0.05; Table 2).

These differences were limited to specific time points and were not accompanied by consistent changes across the evaluation period. Most chemical variables demonstrated wide inter-individual variation and did not show consistent patterns across the entire evaluation period. Glucose concentrations did not differ between groups or over time in either joint (*p* > 0.05).

### 3.4 Cytological analysis

Cytological parameters remained stable across dental severity groups and time points. Mean total nucleated cell counts and differential leukocyte populations varied among individual horses but did not demonstrate differences over time or between groups in either the ipsilateral or contralateral joint (*p* > 0.05). The high variability observed within groups indicates heterogeneous individual responses rather than a consistent inflammatory pattern associated with dental abnormalities or their correction (Tables 3, 4).

**Table 3:** Mean values and standard deviations (x̄ ± SD) of cytological variables from synovial fluid of the temporomandibular joint ipsilateral to the dental abnormalities in 33 horses before and after dental treatment, divided into mild (*n* = 11), moderate (mod, *n* = 12), and severe (sev, *n* = 10) groups.

Variables	Group	Time points			
		Baseline	30 days	60 days	90 days
Erythrocytes ( × 10^3^ cells/μL)	Mild	48.79 ± 13.57	3.13 ± 3.76	21.79 ± 33.00	136.91 ± 367.49
	Mod	86.63 ± 194.31	32.93 ± 78.79	30.75 ± 80.74	1.02 ± 1.09
	Sev	55.60 ± 94.55	7.62 ± 8.10	77.52 ± 142.41	52.24 ± 68.12
Total nucleated cells (cells/μL)	Mild	650 ± 1533	75 ± 63	217 ± 291	474 ± 782
	Mod	452 ± 475	239 ± 291	337 ± 725	108 ± 96
	Sev	341 ± 321	532 ± 1343	325 ± 201	197 ± 155
Neutrophils (cells/μL)	Mild	12 ± 25	1 ± 1	83 ± 224	125 ± 337
	Mod	83 ± 226	6 ± 18	5 ± 13	1 ± 2
	Sev	397 ± 1122	81 ± 195	12 ± 23	53 ± 93
Lymphocytes (cells/μL)	Mild	5 ± 9	2 ± 2	1 ± 2	8 ± 21
	Mod	13 ± 23	0.4 ± 1	3 ± 5	0.1 ± 0.3
	Sev	6 ± 10	117 ± 284	0.0 ± 0.0	3 ± 4
Macrophages (cells/μL)	Mild	4 ± 7	0.4 ± 1	0.0 ± 0.0	0.0 ± 0.0
	Mod	0.0 ± 0.0	0.0 ± 0.0	0.0 ± 0.0	0.0 ± 0.0
	Sev	20 ± 49	0.3 ± 0.9	0.0 ± 0.0	0.0 ± 0.0
Eosinophils (cells/μL)	Mild	0.0 ± 0.0	0.0 ± 0.0	0.0 ± 0.0	0.0 ± 0.0
	Mod	0.0 ± 0.0	0.0 ± 0.0	0.0 ± 0.0	0.0 ± 0.0
	Sev	0.0 ± 0.0	0.0 ± 0.0	0.0 ± 0.0	4 ± 10
Synovial membrane cells (cells/μL)	Mild	132 ± 118	50 ± 40	118 ± 98	394 ± 520
	Mod	421 ± 346	253 ± 291	322 ± 729	109 ± 102
	Sev	3,273 ± 7,890	507 ± 1158	314 ± 207	137 ± 68
Cell morphology score	Mild	4 ± 3	3 ± 3	4 ± 3	4 ± 2
	Mod	3 ± 2	3 ± 2	3 ± 2	2 ± 2
	Sev	3 ± 3	4 ± 3	5 ± 4	3 ± 3

No statistically significant differences were detected among groups or time points (*p* > 0.05).

**Table 4:** Mean values and standard deviations (x̄ ± SD) of cytological variables from synovial fluid of the temporomandibular joint contralateral to the dental abnormalities in 33 horses before and after dental treatment, divided into mild (*n* = 11), moderate (mod, *n* = 12), and severe (sev, *n* = 10) groups.

Variables	Group	Time points			
		Baseline	30 days	60 days	90 days
Erythrocytes ( × 10^3^ cells/μL)	Mild	61.95 ± 162.56	265.36 ± 458.70	9.74 ± 26.30	9.52 ± 16.68
	Mod	54.88 ± 119.15	107.00 ± 342.18	9.08 ± 24.45	52.49 ± 123.21
	Sev	57.76 ± 122.32	18.56 ± 34.53	9.67 ± 14.40	396.56 ± 1,159.93
Total nucleated cells (cells/μL)	Mild	3 ± 7	4 ± 7	3 ± 6	8 ± 2
	Mod	393 ± 67	326 ± 42	169 ± 228	261 ± 357
	Sev	318 ± 286	242 ± 272	89 ± 131	113 ± 109
Neutrophils (cells/μL)	Mild	168 ± 541	34 ± 73	19 ± 59	62 ± 164
	Mod	2 ± 5	5 ± 14	0.1 ± 0.3	10 ± 29
	Sev	8 ± 18	11 ± 18	2 ± 4	4 ± 8
Lymphocytes (cells/μL)	Mild	133 ± 420	16 ± 28	0.0 ± 0.0	0.4 ± 1
	Mod	0.0 ± 0.0	5 ± 9	0.0 ± 0.0	4 ± 14
	Sev	18 ± 26	70 ± 220	0.0 ± 0.0	0.0 ± 0.0
Macrophages (cells/μL)	Mild	164 ± 543	2 ± 6	0.4 ± 1	0.0 ± 0.0
	Mod	0.0 ± 0.0	1 ± 5	0.0 ± 0.0	0.0 ± 0.0
	Sev	3 ± 9	0.4 ± 1	0.0 ± 0.0	0.0 ± 0.0
Eosinophils (cells/μL)	Mild	0.0 ± 0.0	0.0 ± 0.0	0.0 ± 0.0	0.0 ± 0.0
	Mod	0.0 ± 0.1	0.0 ± 0.0	0.0 ± 0.0	0.0 ± 0.0
	Sev	6 ± 19	0.0 ± 0.0	0.0 ± 0.0	0.0 ± 0.0
Synovial membrane cells (cells/μL)	Mild	2,420 ± 6,239	357 ± 606	104 ± 149	749 ± 1855
	Mod	318 ± 616	169 ± 195	154 ± 224	248 ± 319
	Sev	188 ± 226	123 ± 142	86 ± 128	91 ± 106
Cell morphology score	Mild	2 ± 2	2 ± 2	1 ± 2	3 ± 3
	Mod	3 ± 3	2 ± 3	2 ± 3	2 ± 2
	Sev	4 ± 3	4 ± 4	1 ± 2	2 ± 3

No statistically significant differences were detected among groups or time points (*p* > 0.05).

### 3.5 Occlusal adjustment

Occlusal adjustment was effective in correcting the majority of dental abnormalities identified at baseline, including sharp enamel points, focal malocclusions, and occlusal irregularities across all severity groups. Improvement in occlusal balance and masticatory surface alignment was achieved not only in horses classified with mild and moderate dental conditions, but also in those with severe abnormalities.

## 4 Discussion

This study evaluated whether dental abnormalities of different severities influence the physicochemical and cytological characteristics of the TMJ synovial fluid in horses, and whether occlusal adjustment modifies these parameters over time. The hypothesis was only partly supported by the results. The idea that more severe dental conditions would produce more pronounced synovial changes was not confirmed, since most variables remained stable across the severity groups, aside from isolated differences in density, total protein, and pH in the moderate group. The assumption that dental treatment would gradually normalize synovial characteristics was also not supported, as most parameters did not change over time. In addition, changes were not observed in the joint ipsilateral to the dental alterations, indeed, the improvement in fluid appearance in severe cases occurred on the contralateral side. These findings indicate that the TMJ shows considerable stability in its synovial profile, even in the presence of dental abnormalities and after occlusal adjustment.

The clinical relevance of dental procedures for improving equine welfare and masticatory comfort is well documented (19), and several authors have suggested that malocclusions may contribute to discomfort or inflammatory changes in the TMJ (20). While clinical signs such as dysmastication, dropping feed, reluctance during work, and abnormal head posture have been historically associated with TMJ disorders (21, 22), the present results indicate that such clinical signs do not necessarily correspond to measurable inflammatory alterations in TMJ synovial fluid. These findings align with previous studies reporting limited synovial fluid reactivity in the TMJ following dental procedures or altered jaw biomechanics (23, 24).

The stability observed in most physicochemical parameters supports earlier reports describing minimal synovial variation following dental interventions (23). In particular, the lack of change in total nucleated cell counts contrasts with the original expectations but supports previous observations that TMJ synovial fluid demonstrates low inflammatory responsiveness to short-term mechanical alterations (24). The large standard deviations observed across several variables reflect substantial inter-individual variability within groups, and these findings should therefore be interpreted with caution. These results suggest that, unlike other equine joints where synovial fluid readily reflects inflammatory or degenerative conditions (11), the TMJ may possess distinct physiological or biomechanical buffering capacities.

The increase in synovial fluid volume observed in the ipsilateral TMJ of horses with moderate dental abnormalities may suggest a transient increase in synovial production. This finding could be associated with altered mandibular loading patterns prior to treatment or a physiological adjustment following correction. As communication between the discotemporal and discomandibular compartments has been reported to be uncommon (3), the synovial fluid collected in this study is likely to primarily reflect changes within the discotemporal compartment. However, it is important to consider that arthrocentesis does not necessarily allow complete recovery of synovial fluid, and therefore volume measurements should be interpreted with caution. Variability in arthrocentesis technique has been reported to influence aspirated volumes in equine joints (25), and the observed differences may reflect a combination of biological variation and procedural factors rather than a definitive biological response.

The clearer appearance of contralateral synovial fluid in horses with severe dental abnormalities at 60 and 90 days may reflect improved masticatory symmetry after occlusal correction. Mild turbidity in TMJ synovial samples can be associated with minimal blood contamination (26), and the progressive clarity observed may reflect decreased microtrauma or improved technical consistency across sampling sessions.

In the contralateral joint, the moderate group showed isolated increases in synovial fluid density and total protein concentration at 30 days compared with the other groups. Although these variables are known to correlate positively in equine synovial fluid (26), the changes observed here were not consistent across the remaining time points and did not follow a pattern suggestive of inflammation or sustained biochemical alteration. These findings are more likely to reflect individual variability in synovial composition or transient physiological fluctuations rather than a direct effect of the dental condition or its correction. Regarding pH, the moderate group presented higher values at baseline and a reduction at 90 days compared with the earlier assessments. Despite these differences, the values did not remain altered over consecutive evaluations, and no concurrent changes in other inflammatory markers were observed. Previous studies have reported limited diagnostic value of pH in equine TMJ synovial fluid (15, 23), which is consistent with the lack of a clear physiological pattern in the present data. Similar difficulty in identifying diagnostically useful synovial markers was reported by Pereira et al. (24), who found limited changes in inflammatory mediators.

This study presents some limitations. The synovial fluid sampling was restricted to the discotemporal compartment of the TMJ. Although some studies describe a lack of physiological communication between the discotemporal and discomandibular compartments (3, 11, 27), one study describes the communications of these compartments (14). This ongoing debate warrants a cautious interpretation of compartment-specific findings, as synovial alterations in one compartment may not fully reflect changes occurring in the other. In addition, the stable synovial profile observed in this study may reflect adaptive or compensatory mechanisms associated with chronic dental conditions.

Another limitation of this study is the absence of a control group of horses without dental abnormalities. As all included animals presented some degree of dental disorder, it was not possible to establish synovial fluid reference values for a truly healthy TMJ. This limitation restricts the interpretation of the findings, as comparisons were limited to varying degrees of pathology rather than normal vs. abnormal conditions. In addition, the relatively small subgroup sizes may have reduced the ability to detect subtle differences between groups. Future studies including a larger number of animals and clinically normal controls are needed to establish baseline parameters and improve the interpretation of TMJ synovial fluid findings.

Also, the horses included in this study had longstanding dental abnormalities, and it is plausible that initial synovial responses to orodental imbalance reduce over time as biomechanical compensation develops. Consequently, acute, or early-stage dental disorders may induce more pronounced synovial changes that were not captured in this study. Longitudinal studies focusing on acute dental alterations, early intervention, and simultaneous assessment of both TMJ compartments may help clarify the temporal dynamics of synovial responses to dental pathology. In addition, the use of more sensitive biomarkers, such as inflammatory mediators (e.g., interleukins, tumour necrosis factor-α, prostaglandin E2) and matrix metalloproteinases, may allow detection of subtle changes not identified through conventional physicochemical and cytological analysis.

From a clinical perspective, the present findings raise the hypothesis that TMJ synovial fluid analysis may have limited sensitivity for detecting changes associated with dental abnormalities or occlusal correction in horses. Although this was not directly tested, our findings suggest that clinicians should not rely only on synovial fluid analysis when assessing dental-related TMJ dysfunction, and this hypothesis warrants further investigation in future studies.

## 5 Conclusions

The equine temporomandibular joint demonstrated substantial biochemical stability despite dental malocclusions and their correction. These findings indicate that routine dental abnormalities appear to produce limited detectable synovial alterations in the discotemporal compartment, reinforcing the need for TMJ-specific reference values and for future studies exploring both joint compartments and more sensitive biomarkers.

## Data Availability

The raw data supporting the conclusions of this article will be made available by the authors, without undue reservation.

## References

[1] Easley J Tremaine WH. “Dental and oral examination,” *Equine Dentistry*. Saint Louis, MO: W.B. Saunders (2011). 185–98. doi: 10.1016/B978-0-7020-2980-6.00012-X

[2] Carmalt JL Kneissl S Rawlinson JE Zwick T Zekas L Ohlerth S . Computed tomographic appearance of the temporomandibular joint in 1018 asymptomatic horses: a multi-institution study. Vet Radiol Ultrasound. (2016) 57:237–45. doi: 10.1111/vru.12334 26773281

[3] Pimentel KL Carmalt JL. The frequency of communication between the synovial compartments of the equine temporomandibular joint: a contrast-enhanced computed comographic assessment. Front Vet Sci. (2021) 8:753983. doi: 10.3389/fvets.2021.753983 34760960 PMC8573115

[4] Baker GJ. Equine temporomandibular joints (TMJ): morphology, function, and clinical disease. Proc Am Assoc Equine Pract. (2002) 48:442–7.

[5] Carmalt JL. Equine poor performance: the logical, progressive, diagnostic approach to determining the role of the temporomandibular joint. J Am Vet Med Assoc. (2024) 262:397–404. doi: 10.2460/javma.23.09.0513 38016273

[6] Jasiński T Turek B Kaczorowski M Brehm W Skierbiszewska K Domino M. Equine temporomandibular joint diseases: a systematic review. Equine Vet J. (2025) 57:1427–45. doi: 10.1111/evj.14462 39861936 PMC12508285

[7] Ramzan PHL. The temporomandibular joint: component of clinical complexity. Equine Vet J. (2006) 38:102–4. doi: 10.2746/042516406776563323 16536375

[8] Mahaffey E. “Synovial fluid,” *Diagnostic Cytology and Hematology of the Horse*. London: Mosby (2002). 163–170. doi: 10.1016/B978-0-323-01317-8.50013-4

[9] Sterkenburgh TR Hartl B Peham C Nowak M Kyllar M Kau S. Temporomandibular joint biomechanics and equine incisor occlusal plane maintenance. Front Bioeng Biotechnol. (2023) 11:1249316. doi: 10.3389/fbioe.2023.1249316 37799811 PMC10549988

[10] Bouloux GF. The use of synovial fluid analysis for diagnosis of temporomandibular joint disorders. Oral Maxillofac Surg Clin N Am. (2018) 30:251–6. doi: 10.1016/j.coms.2018.03.001 29861340

[11] May KA Moll HD Howard RD Pleasant RS Gregg JM. Arthroscopic anatomy of the equine temporomandibular joint. Vet Surg. (2001) 30:564–71. doi: 10.1053/jvet.2001.28438 11704953

[12] Speirs VC. Clinical Examination of Horses. Philadelphia: W.B. Saunders Company (1997). 358.

[13] Hague BA Honnas CM Simpson RB Peloso JG. Evaluation of skin bacterial flora before and after aseptic preparation of clipped and nonclipped arthrocentesis sites in horses. Vet Surg. (1997) 26:121–5. doi: 10.1111/j.1532-950X.1997.tb01474.x 9068162

[14] Rosenstein DS Bullock MF Ocello PJ. Clayton HM. Arthrocentesis of the temporomandibular joint in adult horses. Am J Vet Res. (2001) 62:729–33. doi: 10.2460/ajvr.2001.62.729 11341393

[15] Fonseca FA Zambrano RS Dias GMB Lima EMM Alves GES Godoy RF. Características fisicoquímicas e citológicas do líquido sinovial da articulação temporomandibular em eqüinos. Pesq Vet Bras. (2009) 29:829–33. doi: 10.1590/S0100-736X2009001000009

[16] Steel CM. Equine synovial fluid analysis. Vet Clin North Am Equine Pract. (2008) 24:437–54. doi: 10.1016/j.cveq.2008.05.004 18652964

[17] Salem SE Townsend NB Refaai W Gomaa M Archer DC. Prevalence of oro-dental pathology in a working horse population in Egypt and its relation to equine health. Equine Vet J. (2017) 49:26–33. doi: 10.1111/evj.12533 26526823

[18] Dixon P du Toit N Dacre I. “Equine dental pathology,” *Equine Dentistry*. Saint Louis, MO: W.B. Saunders (2011). 129–147 doi: 10.1016/B978-0-7020-2980-6.00010-6

[19] Carmalt JL. Evidence-based equine dentistry: preventive medicine. Vet Clin North Am Equine Pract. (2007) 23:519–24. doi: 10.1016/j.cveq.2007.03.002 17616326

[20] Carmalt JL. Equine temporomandibular joint (TMJ) disease: fact or fiction? Equine Vet Educ. (2014) 26:64–5. doi: 10.1111/eve.12103

[21] Carmalt JL Wilson DG. Arthroscopic treatment of temporomandibular joint sepsis in a horse. Vet Surg VS. (2005) 34:55–8. doi: 10.1111/j.1532-950x.2005.00010.x 15720597

[22] Jørgensen E Christophersen MT Kristoffersen M Puchalski S Verwilghen D. Does temporomandibular joint pathology affect performance in an equine athlete? Equine Vet Educ. (2015) 27:126–30. doi: 10.1111/eve.12268

[23] Zambrano RS Fonseca FA Moraes JM Dias GMB Alves GES Lima EMM . Aspectos fisicoquímicos e citológicos do líquido sinovial da articulação temporomandibular de equinos em diferentes idades. Pesq Vet Bras. (2011) 31:926–32. doi: 10.1590/S0100-736X2011001000015

[24] Pereira TP Staut FT Machado TSL Brossi PM Baccarin RYA Michelotto PV. Effects of the oral examination on the equine temporomandibular joint. J Equine Vet Sci. (2016) 43:48–54. doi: 10.1016/j.jevs.2016.04.091

[25] van Pelt RW. Interpretation of synovial fluid findings in the horse. J Am Vet Med Assoc. (1974) 165:91–5. doi: 10.2460/javma.1974.165.01.91 4134829

[26] Barnabé PA Cattelan JW Cadioli FA Godoy RF. Características físico-químicas e citológicas do líquido sinovial da bainha tendínea digital de eqüinos. Arq Bras Med Vet Zootec. (2005) 57:288–94. doi: 10.1590/S0102-09352005000300002

[27] Rodríguez MJ Agut A Gil F Latorre R. Anatomy of the equine temporomandibular joint: study by gross dissection, vascular injection, and section. Equine Vet J. (2006) 38:143–7. doi: 10.2746/042516406776563378 16536383

